# Social Conformity in Autism

**DOI:** 10.1007/s10803-018-3809-1

**Published:** 2018-11-14

**Authors:** Stephanie C. Lazzaro, Laura Weidinger, Rose A. Cooper, Simon Baron-Cohen, Christina Moutsiana, Tali Sharot

**Affiliations:** 1 Affective Brain Lab, Experimental Psychology, University College London, London, UK; 2 Institute of Cognitive Neuroscience, University College London, London, UK; 3 Berlin School of Mind and Brain, Humboldt University of Berlin, Berlin, Germany; 4 Present Address: Department of Psychology, Boston College, Boston, USA; 5 Department of Psychology, University of Cambridge, Cambridge, UK; 6 Present Address: Psychology, School of Social Sciences, University of Westminster, London, UK; 7 Division of Psychology and Language Sciences, University College London, 26 Bedford Way, London, WC1H 0AP UK

**Keywords:** Autism, Social cognition, Conformity, Memory

## Abstract

Humans are extremely susceptible to social influence. Here, we examine whether this susceptibility is altered in autism, a condition characterized by social difficulties. Autistic participants (N = 22) and neurotypical controls (N = 22) completed a memory test of previously seen words and were then exposed to answers supposedly given by four other individuals. Autistic individuals and controls were as likely to alter their judgements to align with inaccurate responses of group members. These changes reflected both temporary judgement changes (public conformity) and long-lasting memory changes (private conformity). Both groups were more susceptible to answers believed to be from other humans than from computer algorithms. Our results suggest that autistic individuals and controls are equally susceptible to social influence when reporting their memories.

Humans are highly influenced by their social environment and frequently conform to the judgements, opinions, beliefs, and actions of others (Asch 1955; Haun et al. 2013; Izuma and Adolphs 2013; Smith et al. 2007). This tendency is so powerful that people’s perceptions, preferences, and memories often change when they contradict a larger consensus, even in cases where the majority is wrong (Meade and Roediger 2002; Hirst and Echterhoff 2012; Lewandowsky et al. 2012). A reduced tendency to conform would make individuals “immune” to some of the dangers of social conformity, such as blindly relying on groups with inaccurate information or falling prey to propaganda. However, reduced social conformity could also cause individuals to feel isolated and separated from their social environment. A decrease in social conformity could also result in learning impairments, as learning is often accomplished in a social setting allowing individuals to automatically benefit from others’ knowledge.

Because Autism Spectrum Disorders (henceforth autism) are characterized both by learning impairments (O’Brien and Pearson 2004) as well as social isolation and disengagement (Orsmond et al. 2013; Howlin and Moss 2012), it has been suggested that autism may be associated with reduced social conformity (Marsh et al. 2013).

To our knowledge only one study to date examined this suggestion in adults (Bowler and Worley 1994; but see Maras and Bowler 2011, 2012; North et al. 2008 for investigation of related constructs). That study compared a small sample of autistic^1^ adults to neurotypical controls and found similar conformity rates in both groups (Bowler and Worley 1994). However, a study examining children (Yafai et al. 2014) found that autistics conformed less than neurotypical controls, with higher scores on a scale measuring autistic traits negatively associated with social conformity. A similar association of autistic traits and lower sensitivity to peer influence was also reported in a study assessing adolescents within the domain of prosocial behaviour. However, this effect was only observed for ‘antisocial’ peer influence (when peers did not cooperate), and did not hold up for ‘prosocial’ peer influence (when peers did cooperate) (Van Hoorn et al. 2017). At the group level, this study found autistic adolescents exhibited equivalent social conformity to neurotypical controls (Van Hoorn et al. 2017). Given the very limited literature on the topic and the diversity of the populations studied (e.g., in terms of age) it is unclear whether autism is indeed associated with changes to social conformity.

Moreover, when examining social conformity, it could be beneficial to dissociate public conformity and private conformity. Making socially conforming judgements that follow the crowd is not always accompanied by an internal change to beliefs (Haun et al. 2013; Kelman 1961; Meade and Roediger 2002; Smith et al. 2007). At times, individuals will select to overtly conform purely for social gain and approval (i.e., to avoid rejection, ridicule or punishment) but privately maintain their original beliefs (Asch 1955; Kelman 1961; Smith et al. 2007). This type of conformity is often referred to as ‘public conformity’. ‘Private conformity’, on the other hand, describes a situation in which individuals internalize the opinion of the crowd resulting in a long-lasting, persistent change to beliefs, preferences, and memories (Edelson et al. 2011). These two types of conformity are associated with different cognitive and neural processes (Edelson et al. 2011), which have often been confounded in research. Autistic individuals could display one type of conformity but not the other, neither type, or both. In particular, public conformity involves a desire to fit in (or feeling pressured to do so), while private conformity involves putting greater weight on the group’s opinion than our own, resulting in persistent modification of beliefs, preferences, and memories. Dissociating between private and public conformity effects in autism may thus be informative for understanding the mechanisms underlying some of the social challenges associated with this population.

To that end, we adapted a well-studied conformity task (Edelson et al. 2011, 2014a, 2014b) that involves multiple memory tests to quantify judgement modification following the introduction of social influence and its subsequent removal. In this context, private conformity is considered a case in which individuals persist in favouring the option endorsed by the group even after social influence is removed. Public conformity is thought not to involve internalization and is thus expected to reverse when the reliability of the socially conveyed information abates (Asch 1955; Kelman 1961; Smith et al. 2007).

We specifically wanted to test how information endorsed by other humans influences autistic individuals, while controlling for the ability to extract information from others or attend to other people. Thus, we used a task in which participants receive information from others that are not physically in the room with them at the time of influence (Edelson et al. 2011, 2014a, 2014b). This is analogous to asking whether autistic individuals would be influenced by ratings on Amazon that contradict their own, for example.

Our task examines social conformity in the domain of memory. It has been suggested that autism may be associated with specific memory impairments such as reduced ability to monitor memory source (Craig et al. 2016; Maras and Bowler 2014; Bowler et al. 2004; Cooper et al. 2016) and increased reliance on familiarity (Bowler et al. 2000), though recollection is not always found to be impaired in autism (e.g., North et al. 2008; Maras and Bowler 2012). If autistic individuals do have difficulties in memory, they may rely on others’ recollections to fill their own gaps. Our paradigm enables us to quantify baseline differences in memory separately from memory conformity. Furthermore, to be able to differentiate effects of social influence from non-social influence, we manipulate the source of information presented to the participants as either coming from other participants (social) or from computer algorithms (non-social).

## Methods

### Participants

Sample size was determined based on past studies (Edelson et al. 2011, 2014a, 2014b). 33 neurotypical control (NC) adults with no history of psychiatric or neurological conditions and 23 autistic adults (autism) participated in the study. One NC participant was excluded due to neither understanding nor following task instructions, three participants were excluded due to technical faults (one autism, two NCs), and eight NC participants were excluded as a result of debriefing responses indicating that they may have guessed the ‘group’ responses were fabricated. These eight participants did not differ from included control participants on intelligence (WTAR standardized scores, t(24) = .074, p = .942), or demographics: age, t(28) = .338, p = .738, gender, t(28) = 1.269, p = .215, or AQ scores, t(24) = 1.393, p = .176. Note, that the 24% exclusion rate due to the suspicion of deception is in line with previous studies of social conformity (Edelson et al. 2011, 2014a; Stang 1976; Stricker et al. 1967; Mori and Arai 2010).

The resulting final sample of 44 participants included 22 autistic individuals (10 female) and 22 NCs (14 female). The groups were matched at the group level on age (autism mean = 24.4, SE = 1.64; NC mean = 23.23, SE = 1.22; t(42) = .577, p = .567) and verbal IQ as predicted from Wechsler Test of Adult Reading (WTAR) scores (autism mean = 108.53, SE = 1.62; NC mean = 111.83 SE = 1.63; t(33) = 1.44, p = .159, 5 NC participants and 4 autistic participants failed to complete this test).

Autistic participants were recruited via the Autism Research Centre, Cambridge (N = 18) and local Autism/Asperger Syndrome services and support groups (N = 4). All autistic participants met a diagnosis for High Functioning Autism (HFA, N = 7) or Asperger Syndrome (AS, N = 15), and received their diagnosis following specialist assessment by a qualified clinician. To support diagnosis, Autism Spectrum Quotient (AQ; Baron-Cohen et al. 2001) scores for all autistic participants were obtained from the Cambridge Autism Research Database or via administration of the questionnaire (mean = 33.77, SE = 1.58) and were significantly higher than NC participants’ AQ scores (mean = 16.44, SE = .89; t(38) = − 8.994, p = .004, three NC participants failed to complete this test). Woodbury-Smith et al. (2005) found that an AQ score greater than or equal to 26 resulted in the highest proportion of their sample being correctly classified as having a diagnosis of autism. All autistic participants in our sample had an AQ score above 25 and all NC participants had a score below 25. All neurotypical controls were recruited via the University online participant pool. Participants gave written informed consent prior to participating and were compensated for their time. The study was approved by the University Research Ethics Committee.

### Stimuli

Two hundred and fifty emotionally neutral nouns, 4–7 letters in length, randomly selected from the Bristol Norms (Stadthagen-Gonzalez and Davis 2006) were used in the experiment. Presentation order was randomized for each participant.

### Procedure

Our methods and analysis followed a well-studied procedure that helps dissociate between public and private conformity (Edelson et al. 2011, 2014a, 2014b). We made three changes to the procedure: (i) the stimuli used were words (while the original task uses a film), (ii) the delay between the different tests was shorter in order to complete testing in 1 day and (iii) group members were represented on the screen as cartoon figures instead of photographs.

**Fig. 1:**
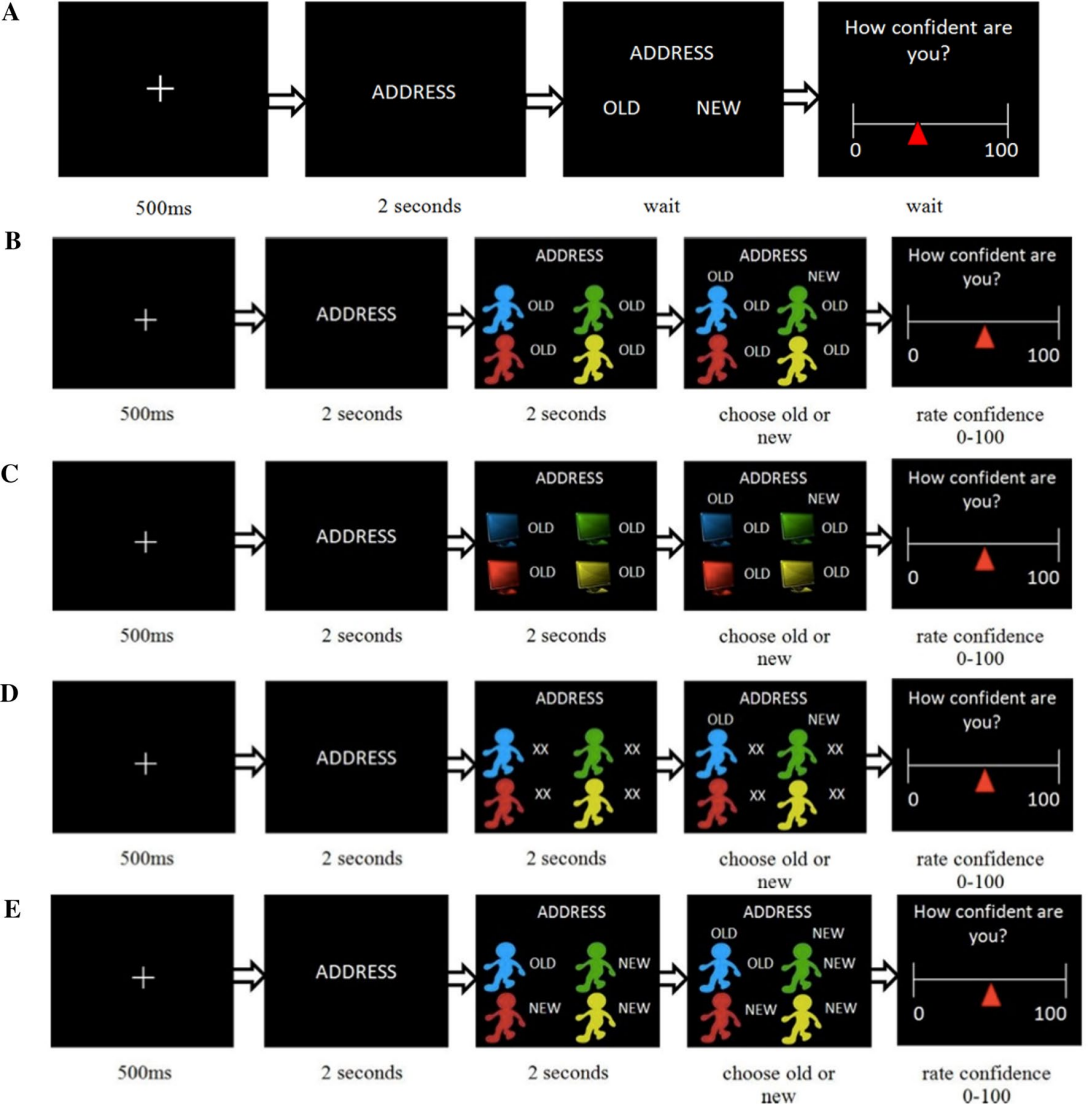
Task. Participants viewed words in groups of five participants and subsequently performed three memory tests individually. **a** An example trial of Test 1, which served to assess the participants’ initial memory and confidence before the manipulation administered in Test 2. On each trial participants viewed a word and were asked to indicate whether they had seen the word in the first part of the study (old) or not (new) and indicate their confidence in their answer. **b**–**e** Test 2 consisted of trials of four different conditions. In all conditions a word was presented for 2 s, followed by either the fabricated co-observers’ answers for 2 s or the computer answers. Participants then submitted their answer (old or new) and confidence level. **b** In the Social Manipulation trials all co-observers’ answers were incorrect. **c** In the Non-Social Manipulation trials all computer answers were incorrect. **d** In the No-Manipulation condition ‘XX’ was displayed instead of co-observers’ answers or instead of computer answers (the latter not shown in the figure). **e** In the Credibility Condition variable patterns of co-observers’ answers (or computer answers) were displayed. **a** Test 3 was the same as Test 1 and served to identify memory errors that persisted after the manipulation was removed

The experiment protocol (Fig. 1) spanned four phases over approximately 4 h and included a word encoding phase, followed by three recognition memory tests: baseline (Test 1), followed by a 30-min break, manipulation (Test 2), followed by a 45-min break, and recovery (Test 3). All phases were programmed and run using MATLAB (Mathworks Inc.) and the Cogent 2000 toolbox. Participants took part in groups of five unacquainted individuals including one autistic participant, one or two NC participants, and two or three confederates. None of the participants included in the sample indicated suspicion that any of the group members were confederates. All groups included females and males, and the proportion between females and males was not fixed but differed for each group. Participants were not given any information about the other participants during the recruitment process nor during the experiment itself. Controls were not aware the study included autistic individuals, and autistic participants were not informed whether other participants in the group were autistic. Participants were told they might encounter the other members of the group during the breaks and were instructed not to discuss the experiment with the other participants at that time. They did not know whether they would also meet again at the end of the study.

#### Encoding

Participants sat together in a room and completed informed consent and demographics forms. Participants were told they would be shown a series of words on a single screen visible to all group members and that they should attend to all of the words in preparation for a later memory test. In addition, participants were told that some words would appear twice in a row and were instructed to write down the first letter of any word that appeared twice in a row, but not to write down anything else. These duplicate words were included to maintain participants’ attention during encoding. Instructions for the task were followed by 125 words, each of which appeared for 2.5 s followed by a fixation cross for 500 ms. The five duplicate words were later included as practice trials for each memory test.

#### Baseline (Test 1)

Immediately following the encoding stage, participants were taken to adjacent individual cubicles to complete the rest of the experiment. This first recognition memory test served to assess the participants’ *baseline memory accuracy and confidence* before the manipulation phase and was comprised of the presentation of 120 words that had been shown during the encoding stage (‘old words’) and 120 new, unstudied words in random order (Fig. 1a). The task started with 10 practice trials of 5 old (the duplicated words) and 5 new words. Each word was displayed alone for 2 s before the options ‘new’ and ‘old’ were presented on either side of the screen. The lateral position of ‘old’ and ‘new’ reversed randomly to ensure that participants thought about each response and participants were warned this would happen. Participants were asked to indicate whether a word was ‘old’ or ‘new’ using a key press (‘g’ for the option on the left, ‘h’ for the option on the right). Participants then rated their confidence in their response on a scale from 0 to 100. Judgement and confidence responses were self-paced. The test was followed by a 30-min break during which participants could relax. During this time participants were told that the experimenter was ‘uploading’ each participant’s Test 1 responses for the next phase.

#### Manipulation (Test 2)

Here an attempt was made to influence participants’ answers (Fig. 1b). Manipulations were performed in two blocks: a social condition (120 trials) and a computer condition (120 trials) with order counterbalanced across participants. Participants answered all 240 memory questions as in Test 1, but this time before answering, they were presented with the Test 1 answers supposedly given by their four fellow co-participants (social condition) or by four computer programs (computer condition). Each word appeared alone for 2 s followed by the answers from four co-observers or four computer programs for 2 s before participants could respond. Answers remained on the screen until the participant responded and then participants indicated their confidence in their response. For the social condition, participants were instructed that the responses were anonymous answers from the other members of their group in Test 1. For the non-social condition, participants were told four computer programs had searched a database of words and chosen the answer most likely to be correct according to an algorithm (no detail on the nature of the algorithm was revealed), and that the accuracy of this algorithm was comparable to the average human participant. Co-observers and computer programs were represented with cartoon drawings. Participants were instructed that the answers of co-observers and computer programs could be used to assist their retrieval but that they were ultimately required to answer according to their own memory. The co-observer and computer program answers were pseudo-randomly allocated into three different categories as follows (see Fig. 1b–e).

##### Manipulation Trials (40 Social Trials, 40 Computer Trials)

The function of these trials is to estimate the effect of social/non-social influence. The trials included words for which participants had correctly answered ‘old’ or ‘new’ during Test 1 and their confidence was within 20–90% of their range of confidence ratings. This confidence range was increased to a maximum of 15–95% if the former range did not generate 80 trials in total. Crucially, for these manipulation trials, all four responses from co-observers (Fig. 1b) or computers (Fig. 1c) were incorrect. Previous studies show that unanimous responses of group members elicit the strongest and most consistent conformity (Asch 1955; Edelson et al. 2011, 2014a).

##### No Manipulation Control Trials (10 Trials in Social Block, 10 Trials in Computer Block)

The function of these trials is to estimate baseline forgetting. The same criteria were used to select stimuli as for the Manipulation trials. Here, the letters ‘XX’ were presented instead of a response from co-observers (Fig. 1d) or computers, thus providing participants with no new information.

##### Credibility Trials (70 Social Trials, 70 Computer Trials)

The function of these trials was to reduce the likelihood that participants would suspect that responses were fabricated. The stimuli presented here included all remaining stimuli presented during Test 1. ‘Responses’ of the co-observers or computer programs were determined as follows: Test 1 trials that had a confidence below the threshold for manipulation trials were presented with responses that showed a ‘split-decision’, (i.e., 2 said old and 2 said new), and the remaining trials showed that 3 or all 4 co-observers (Fig. 1e) or computer responses were in agreement with the participant’s response during Test 1, regardless of whether it was correct or not.

Following Test 2, there was a 45-min break during which participants completed a separate decision-making task in which they rated food and drink preferences and obtained a highly rated food or drink item.

#### Recovery (Test 3)

Here, following Edelson et al. (2011), we attempted to retroactively dissociate private and public conformity. Before beginning Test 3, participants were informed that the answers given by the co-observers and computer programs during the previous session had actually been determined randomly, rendering these answers uninformative. The participants were then requested to complete the same memory test again (Test 3) based on their memory of the words, following the same procedure as Test 1. Items that the participants answered correctly in Test 1 but incorrectly in both Tests 2 and 3 are considered items that reflect long lasting changes to memory (‘private’ conformity). Items that the participants answered correctly in Test 1, incorrectly in Test 2, but then reverted back to the original correct answer when social influence was lifted in Test 3, are considered items that reflect ‘public’ conformity.

Following Test 3, participants completed an online debriefing form and three questionnaires:

1. Liebowitz Social Anxiety Scale (LSAS, Liebowitz 1987) which includes 24 items, has a Chronbach’s alpha of .95 (Liebowitz 1987; Baker et al. 2002) and high reliability in neurotypical individuals (Heimberg et al. 1999; Fresco et al. 2001; Masia-Warner et al. 2003; von Glischinski et al. 2018) and autistics (Baron-Cohen et al. 2001). The LSAS scores correlate with AQ scores (Baron-Cohen et al. 2001).
2. Mehrabian Social Conformity Scale (Mehrabian and Stefl 1995), which includes 11 items, has an alpha of .77 (Mehrabian and Stefl 1995) and high reliability (Mehrabian and Stefl 1995; Wu and Chang 2012; Vučković et al. 2010).
3. Wechsler Test of Adult Reading (WTAR, Wechsler 2001), which includes 50 items, has been co-normed with the Wechsler Adult Intelligence Scale (WAIS-III) and has high reliability in neurotypical individuals (Mathias et al. 2007; Green et al. 2008) and in autistics (Freeman et al. 1985; Venter et al. 1992).

### Analyses

We calculated the error rates during Test 2 for the four conditions of interest (social manipulation condition, computer manipulation condition, no-manipulation condition in social block, no-manipulation condition in computer block) as number of trials for which the participant gave an incorrect answer in that condition divided by number of trials in that condition. For example, if a participant was incorrect on 20 trials out of the 40 social manipulation trials in Test 2, then their error rate is 0.5 (or 50%) in that condition.

All statistical results in which we compare groups or test for correlations with AQ score (i.e. partial correlations) are reported while controlling for possible difference in baseline memory performance (rather than susceptibility to the manipulation) by subtracting for each participant errors in the specific no-manipulation control trials from errors in the specific manipulation trials and conducting statistical tests on those corrected numbers. For example, if participant’s error rate was 0.5 in the social manipulation condition and 0.4 in the no-manipulation condition in the social block than their score would be 0.5 − 0.4 = 0.1. In addition, all ANOVAs and correlation statistics are reported controlling for gender. As there has been an ongoing debate on whether gender is related to social conformity (Stein et al. 1986; Carr 1998; Cross et al. 2017), as well as the expression of autism symptoms (Sipes et al. 2011; Thompson et al. 2003; Lai et al. 2015) we decided to include gender as a covariate in our analyses.

In Test 3 we calculated persistent errors that were due to the manipulation experienced in Test 2. This was achieved by calculating the number of trials participants answered incorrectly on Test 3 and Test 2, separately for trials in which they experienced a social manipulation, computer manipulation, no-manipulation social block, or no-manipulation computer block in Test 2, divided by the number of trials in that condition. For example, if the participant answered 20 of 40 social-manipulation questions incorrectly in Test 2 and out of those still answered 15 incorrectly in Test 3, their persistent error rate for the social manipulation condition is 15/40 = 0.375 (or 37.5%). Trials for which they answered incorrectly in Test 2 but reverted to correct answer in Trial 3 are transient errors. In our example this is equal to 5/40 = 0.125 (or 12.5%).

Again, all statistical analyses are reported while controlling for possible difference in baseline memory accuracy (rather than susceptibility to the manipulation) by subtracting for each participant the persistent error rate in the specific no-manipulation trials from the persistent error rate in the specific manipulation trials and conducting statistical tests on those corrected numbers. For example, if a participant’s persistent error rate was 0.375 in the social manipulation condition and 0.2 in the no-manipulation control condition in the social block then their persistent error score would be 0.375 − 0.2 = 0.175. In addition, all ANOVAs and correlation statistics are reported controlling for gender.

For null results a Bayes Factor (BF) was calculated using JASP software (JASP Team 2018, Version 0.8.5) with a Bayes Factor of 0 < BF < 1 indicating support for the null hypothesis, 1 < BF indicating non-significant support for the alternative hypothesis and 3 < BF indicating significant support for the alternative hypothesis (Kass and Raftery 1995).

## Results

We first tested whether autistic participants would alter their previously correct response to match the incorrect response of the group and whether their conformity rate differed from control participants. Submitting Test 2 errors into a group (autistics/NC) by condition (social manipulation/computer manipulation) ANOVA revealed a main effect of condition (F(1,41) = 4.956, p = .032), with no difference between groups (F(1,41) = .013, p = .909) nor an interaction (F(1,41) = .124, p = .727). The effect of condition was due to the social manipulation inducing greater memory errors than the computer manipulation condition both in autistics and NC participants (autism group: t(21) = 2.137, p < .05, NC group: t(21) = 4.365, p < .001).

In particular, autistic participants conformed to the majority opinion in 66.3% ± 5.6% of social manipulation trials on Test 2, giving an incorrect answer to questions they had answered correctly in baseline Test 1. For controls this was true in 58.9% ± 4.1% of social manipulation trials (no difference between the groups on socially induced errors, t(42) = − .154, p = .878, BF = .300). Errors were also induced by computers in autistic participants (who conformed to the majority incorrect opinion of computer algorithms in 50.7% ± 6.0% of computer manipulation trials) and controls (44.7% ± 5.2%) with no difference in this tendency between groups (t(42) = .225, p = .823, BF = .304). These findings show that autistic participants altered their responses to match false responses of others as often as controls.

**Fig. 2:**
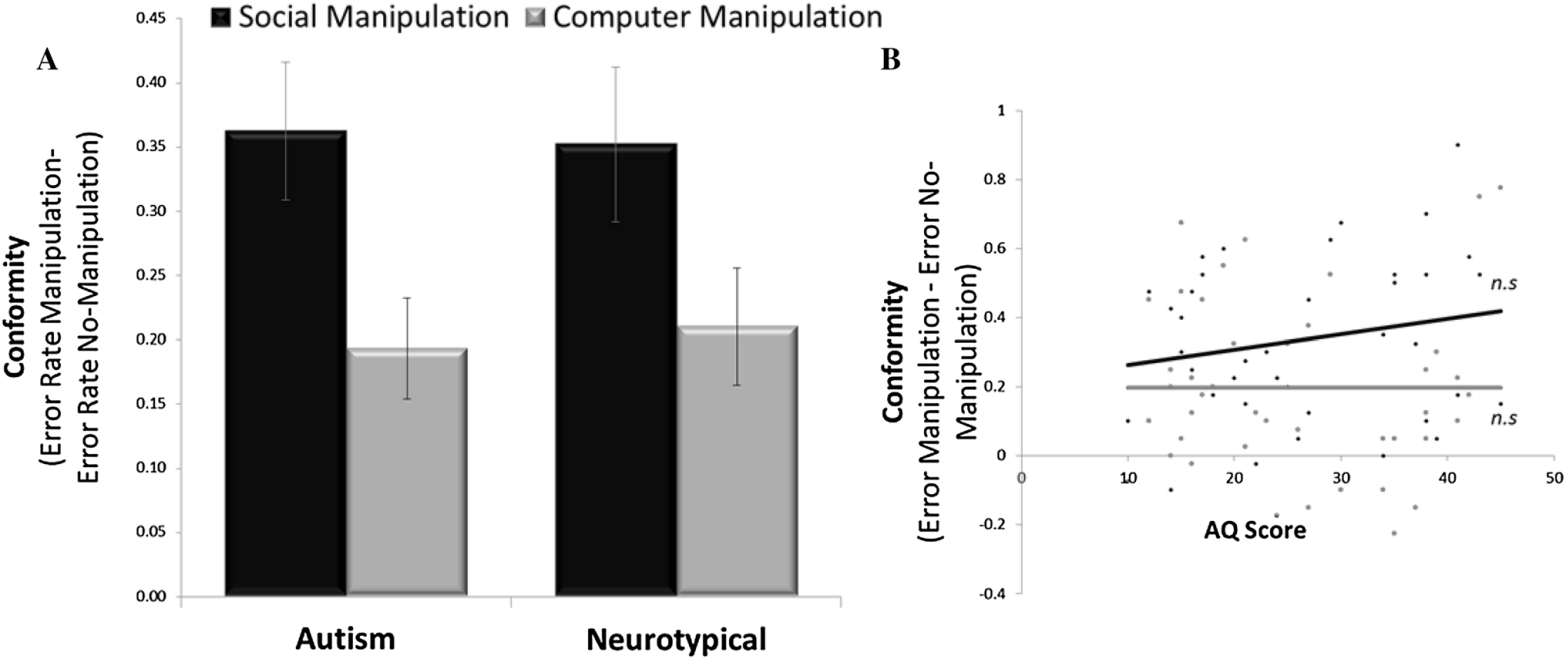
Conformity rates. **a** Both autistic and neurotypical controls were more likely to conform to the erroneous answers believed to be of other participants (social manipulation) than to those believed to have been generated from computers (computer manipulation) on questions they had previously answered correctly. Displayed are the error rates of the manipulation condition after subtracting baseline error rates from the no-manipulation condition (thus controlling for simple forgetting). **b** Conformity rates did not correlate with AQ score. Error bars SEM, *n.s* not significant

No correlation was found between AQ score and conformity in response to social (r(37) = .147, p = .371) or computer (r(37) = − .027, p = .872) manipulation (Fig. 2b) and AQ was not correlated to the Mehrabian Social Conformity Scale, r(37) = .046, p = .783 (Mehrabian and Stefl 1995). Equally, WTAR scores were not related to conformity in the social r(44) = .142, p = .357, BF = .283, or the computer condition r(44) = .185, p = .229, BF = .379.

Next, we tested whether conformity could be explained by differences in baseline memory errors or social anxiety. As a reminder, all statistical tests were conducted while controlling for performance on no-manipulation trials. The latter is subtracted from each participant’s performance on manipulation trials to control for possible difference in memory per se. None of the results change, however, if performance on no-manipulation trials is not controlled for, and there is no significant difference between groups on no-manipulation trials. Specifically, for the *no-manipulation* trials in the social block incorrect answers were given by autistic participants in 30.0% ± 3.0% of the trials (which is significantly lower than their social manipulation error rate: t(21) = − 6.773, p < .001) and in controls on 23.6% ± 3.7% (which is significantly lower than their social manipulation error rate, t(21) = − 9.035, p < .001), with no difference between groups, (t(42) = − 1.334, p = .189, BF = .606). Equally, in the computer block, autistic participants were incorrect in 31.4% ± 4.2% of the no-manipulation trials (which is significantly lower than their computer manipulation error rate, t(21) = − 3.201, p = .004) and controls in 23.6% ± 3.3% (which is significantly lower than their computer manipulation error rate, t(21) = − 4.610, p < .001), again with no difference between groups (t(42) = − 1.459, p = .152, BF = .609). These results indicate no difference in baseline rate of forgetting across groups (see also North et al. 2008; Maras and Bowler 2012).

Consistent with previous literature (Liss et al. 2008) we found that autistics scored higher on the social anxiety scale than controls (autism mean = 67.91, SE = 6.70, NC mean = 26.86, SE = 3.06, t(42) = − 5.571, p < .001). We thus repeated all group comparisons and correlations above in which we compare groups or correlate with AQ score while controlling for social anxiety. This did not alter any of the results. Thus, conformity cannot be explained by differences in baseline memory errors or social anxiety.

Do autistic and control participants make a similar amount of persistent errors? There was no difference between autistic and control participants in the frequency of persistent errors. A group (autistics/controls) by condition (social manipulation/computer manipulation) ANOVA on persistent errors revealed a main effect of condition (F(1,41) = 8.806, p = .005), with no difference between groups (F(1,41) = 1.588, p = .215) nor an interaction (F(1,41) = .314, p = .578). The main effect of condition was characterized by greater persistent errors due to the social manipulation than the computer manipulation.

**Fig. 3:**
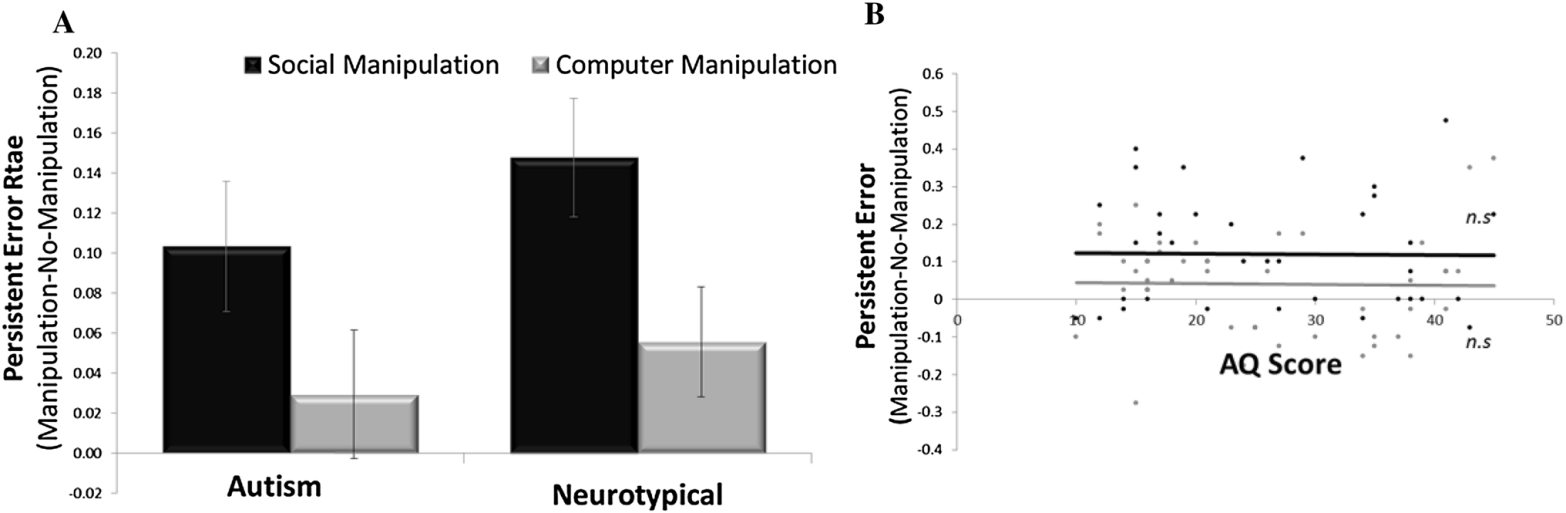
Persistent errors (private conformity). **a** During Test 3, after social/computer influence was lifted, autistic individuals exhibited a similar rate of persistent memory errors on questions for which they previously conformed to the erroneous answers of the group as neurotypical controls. Persistent error rate was greater for trials previously presented in the social manipulation condition than the computer manipulation condition, with no group by condition interaction. Displayed are the persistent error rates of the manipulation condition after subtracting baseline persistent error rates from the no-manipulation condition (thus controlling for simple forgetting). **b** Persistent error rates did not correlate with AQ score. Error bars SEM, *n.s*. not significant

In particular, when social influence was removed (Test 3), autistic participants maintained erroneous memory responses in 28.1% ± 2.3% of social manipulation trials (persistent errors, private conformity). These numbers were not different from the control participants, who maintained erroneous memory responses in 27.0 ± 2.5% of social manipulation trials (persistent social errors, difference between groups t(42) = 1.006, p = .320, BF = .447) (Fig. 3a). Equally, when influence of computer algorithm was removed (Test 3), autistic participants maintained erroneous answers in 22.5% ± 2.8% (persistent errors). These numbers were again not different from the NC participants, who maintained erroneous answers in 19.2% ± 2.7% (persistent computer errors, difference between groups t(42) = .621, p = .538, BF = .348). No correlation was found between AQ score and frequency of persistent errors (r(37) = − .015, p = .925) in the social condition (Fig. 3b) or computer condition (r(37) = − .017, p = .919, Fig. 3b). While there were no differential effects between groups nor interaction, we do note that controls were significantly less likely to exhibit persistent errors due to computer influence than social influence (t(21) = 2.825 p = .010, BF = 4.916), a difference that was not significant in autistics (t(21) = 1.626, p = .119, BF = .695). This suggests that persistent errors due to social influence are comparable in autistics and controls.

We then tested whether persistent errors due to social conformity could be explained by forgetting. In only 17.7% ± 3.0% of trials for which *no* manipulation was previously administered, autistic participants persisted in providing incorrect answers in Test 3 and this number was significantly lower than their social manipulation persistent error rate, (t(21) = 3.178, p < .005). For neurotypical controls the corresponding number was 12.3% ± 1.9%, which is significantly lower than their social manipulation persistent error rate (t(21) = 4.975, p < .0001).

Persistent errors in autistic individuals in the computer manipulation condition seemed to be due to forgetting. In particular, in 19.5% ± 3.1% of trials for which no manipulation was previously administered in the computer condition, participants persisted in providing incorrect answers in Test 3 and this number was not significantly lower than their computer manipulation persistent error rate (t(21) = .923, p < .366). For neurotypical controls the corresponding number was 13.6% ± 2.8%, which was not significantly lower than their computer manipulation persistent error rate, but was trending (t(21) = 2.034, p = .055). As before, repeating all tests above in which we compare groups (or correlate with AQ score) while controlling for social anxiety did not alter the results. In sum, persistent errors due to social conformity cannot be explained by forgetting.

Do autistic and control participants make a similar amount of transient errors? For transient errors (public conformity), the ANOVA revealed no main effects nor interaction (condition: F(1, 41) = 0.180 p = .673; group: F(1, 41) = 0.725, p = .40; interaction: F(1, 41) = 0.850, p = .362). Interestingly, this suggests that the unique effect of social influence (compared to non-social influence) was restricted to persistent, long-term errors.

The effect of condition on transient error rates was comparable between both groups. In particular, when social influence was removed (Test 3), autistic participants reverted back to their correct Test 1 responses in 38.2% ± 4.3% of social manipulation trials (transient errors, public conformity). These numbers were not different from the NC participants, who reverted back to their correct Test 1 responses in 31.8% ± 2.6% of social manipulation trials (transient social errors, difference between groups t(42) = − .985, p = .330, BF = .439). Transient errors due to social manipulation were not correlated with AQ score (r(37) = 0.186, p = .258).

When influence of computer algorithm was removed (Test 3), autistic participants reverted back to their correct Test 1 answers in 28.2% ± 3.9% (transient errors) which was not significantly different from their social manipulation condition (t(21) = − 1.726, p = .099). These numbers were not different from the NC participants, who reverted back to their correct Test 1 answer in 25.5% ± 3.3% (transient computer errors, difference between groups t(42) = − .183, p = .855, BF = .302), which was not different from their social manipulation condition (t(21) = − 1.291, p = .211). Transient errors due to computer manipulation were not correlated with AQ score (r(37) = − 0.027, p = .872). Baseline memory and confidence were comparable between groups (Table 1). These findings show that transient errors due to social influence are comparable in autistics and controls (Table 1).

**Table 1:** Baseline memory and confidence

	ASC M (SE)	NC M (SE)	T, p	Bayes factor
Baseline memory accuracy	67.09 (1.81)	69.24 (1.59)	.894, p = .376	.41
Confidence				
Baseline (T1)	61.67 (11.11)	66.27 (2.26)	− 1.405, p = .168	.65
Social conformity trials (T2)	62.26 (4.35)	60.94 (3.84)	− .227, p = .821	.30
Computer conformity trials (T2)	55.17 (4.22)	57.33 (3.92)	.374, p = .710	.32
Non-conformity trials (T2)	62.72 (3.48)	65.78 (3.64)	.609, p = .546	.35
Transient error trials (T3)	51.05 (3.40)	52.79 (2.82)	.392, p = .697	.32
Persistent error trials (T3)	51.66 (3.64)	53.00 (3.54)	.263, p = .794	.31

Baseline memory accuracy and participants’ confidence did not differ between groups. There was also no time (T1, T2, T3) by group interaction on confidence ratings (F(2,39) = .981, p = .384, BF = .395)

## Discussion

When faced with a situation in which members of the group unanimously express a belief that is contradictory to one’s own, most individuals will alter their judgement to align with the crowd. Our results suggest that this tendency for social conformity is no different in autistic individuals. Indeed, both groups of participants (whether autistic or neurotypical controls) altered their previously correct memory judgements to align with the false memory of the group approximately two times out of three. These cases could not be explained away as simple forgetting because in the absence of this manipulation participants were significantly less likely to alter a correct judgement to an incorrect one. Moreover, information said to be provided by other humans had a greater impact on the beliefs of both autistic individuals and neurotypical controls than information said to be provided by computer programs. In addition, autistic individuals and neurotypical controls displayed similar rates of baseline memory accuracy (in line with North et al. 2008; Maras and Bowler 2012; but see Maras and Bowler 2011) and were equally confident in their memory.

After conforming to the erroneous opinion of others, both neurotypical controls and autistic individuals maintained these new beliefs at a similar rate after being told that the information provided previously was in fact random. These cases are thought to reflect a persistent change to beliefs due to social influence, termed ‘private conformity’, while cases in which participants revert to their original correct beliefs are thought to reflect only transient alteration that are often referred to as ‘public conformity’. The results are consistent with previous findings that autistic adults are as susceptible to misinformation as neurotypical controls (Maras and Bowler 2011), scoring similarly on scales of suggestibility (i.e., personal acceptance of information suggested by another person) (Maras and Bowler 2012; North et al. 2008) and on compliance (i.e., the tendency to agree with another’s suggestion even when privately disagreeing with it) (Maras and Bowler 2012; but see North et al. 2008) which are constructs associated with private and public conformity, respectively.

It is important to note that while our study suggests autistics and controls conform at similar rates, future studies are needed to examine whether they conform for similar reasons. There are different reasons why individuals conform, including the desire to fit in and the desire to be accurate, and it is possible these drives may be more or less prominent in autistic individuals as compared to controls. Moreover, we studied conformity for factual information and it is unknown whether similar findings will be observed for information for which there is no ground truth, such as preferences for food or moral judgements. We also examined social conformity in a situation in which the participants are physically removed from a social context. This design is similar to many previous studies of social conformity (Charpentier et al. 2014; Edelson et al. 2011, 2014a, 2014b; Campbell-Meiklejohn et al. 2010, 2012; Klucharev et al. 2009; Izuma and Adolphs 2013) and to what people experience online, for example via social media. It is possible that autistic individuals would exhibit decreased social conformity when information is conveyed directly by other people, perhaps because of reduced saliency and divergent processing of social cues in those situations (Ashwin et al. 2006; Dawson et al. 2004). That said, our results are consistent with the one study examining social conformity in autistic adults in which all individuals were physically present at time of testing (Bowler and Worley 1994). That study, which was restricted to a small number of adults with Asperger Syndrome (Bowler and Worley 1994), employed the classic Asch conformity task, which examines conformity in relation to perceptual judgements. The authors found that individuals with Asperger Syndrome followed the group’s false judgement to a similar extent as neurotypical controls. The Asch conformity task is typically thought of as reflecting ‘public conformity’ but does not distinguish between public and private conformity.

Another study that employed an “Asch conformity task” but examined children (age = 9.08 ± 1.42 years), concluded autism is associated with a reduction in public conformity (Yafai et al. 2014). Another study examining teenagers reported a more complicated picture with autism symptoms reported to be related to less sensitivity to antisocial peer influence but not prosocial peer influence (Van Hoorn et al. 2017). The different results between these studies and the present experiment may be due to the different age of the autistic participants. While autistic individuals exhibit a tendency to conform, the extent to which they do so differently from neurotypical controls may change with age. It may be that adults with autism have acquired social conformity as a social strategy, whereas autistic children may have yet to develop such a strategy. As our study only tested young adults, further research is required to characterize age-related changes. It should also be noted that criteria for an autism diagnosis have changed over the years (see e.g., Maenner et al. 2014) and one limitation of comparing studies of autism is that the makeup of the resultant diagnostic populations may differ due to diagnostic changes depending on the year the study took place as well as potentially whether the Diagnostic and Statistical Manual of Mental Disorders (DSM) or the World Health Organisation’s International Classification of Diseases (ICD) was used for diagnosis.

Our results suggest that autistic individuals and neurotypical controls are equally susceptible to social influence when reporting their memories. Interestingly, misleading social information led to both transient *and* persistent changes in beliefs, suggesting that in autism social influence also alters not only explicit responses but also internal beliefs.

## Footnotes

1 We use identity-first language and therefore refer to autistic people rather than people with autism, as is commonly requested by autistic people themselves (http://autisticadvocacy.org/about-asan/identity-first-language/).
